# Partial and asymmetrical reproductive isolation between two sympatric tropical shrub species: *Cnidoscolus aconitifolius* and *C. souzae* (Euphorbiaceae)

**DOI:** 10.1002/ece3.10801

**Published:** 2023-12-11

**Authors:** Miguel A. Munguía‐Rosas, Víctor Parra‐Tabla, José M. Rodríguez‐Domínguez

**Affiliations:** ^1^ Laboratorio de Ecología Terrestre, Departamento de Ecología Humana Centro de Investigación y de Estudios Avanzados del Instituto Politécnico Nacional (Cinvestav) Mérida Mexico; ^2^ Departamento de Ecología Tropical Universidad Autónoma de Yucatán Mérida Mexico; ^3^ Centro de Investigación y Asistencia en Tecnología y Diseño del Estado de Jalisco, A.C. Unidad de Biotecnología Vegetal Guadalajara Mexico

**Keywords:** *Cnidoscolus*, heterospecific pollen transfer, post‐zygotic reproductive barriers, pre‐zygotic reproductive barriers, reproductive isolation

## Abstract

Reproductive isolation is conferred by several barriers that occur at different stages of reproduction. Comprehensive reviews on the topic have identified that barriers occurring prior to zygote formation are often stronger than those that occur afterward. However, the overrepresentation of temperate perennial herbs in the current literature precludes any generalization of this pattern to plants that present other life forms and patterns of distribution. Here, we assessed reproductive isolation barriers and their absolute contribution to reproductive isolation and asymmetry in *Cnidoscolus aconitifolius* and *C. souzae*, two closely related tropical shrub species that co‐occur on the Yucatan peninsula. The reproductive barriers assessed were phenological mismatch, pollinator differentiation, pollen–pistil incompatibility (three pre‐zygotic barriers), fruit set failure, and seed unviability (post‐zygotic barriers). Reproductive isolation between the study species was found to be complete in the direction *C. aconitifolius* to *C. souzae*, but only partial in the opposite direction. One post‐zygotic barrier was the strongest example. Most barriers, particularly the pre‐zygotic examples, were asymmetrical and predicted the direction of heterospecific pollen flow and hybrid formation from *C. souzae* to *C. aconitifolius*. Both parental species, as well as the hybrids, were diploid and had a chromosome number 2n = 36. More studies with tropical woody perennials are required to fully determine whether this group of plants consistently shows stronger post‐zygotic barriers.

## Graphical abstract

We assessed reproductive isolation barriers and their absolute contribution to reproductive isolation and asymmetry in *Cnidoscolus aconitifolius* and *C. souzae*, two sister tropical shrub species that co‐occur on the Yucatan peninsula, Mexico.

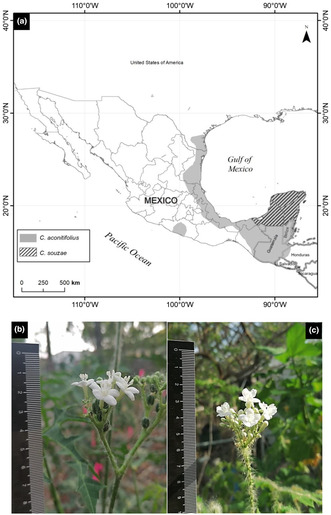

## 1 INTRODUCTION

Reproductive isolation barriers (RIBs) are the biological features of living organisms that limit gene flow among species or populations and confer some degree of reproductive isolation (Coyne & Orr, 2004; Dobzhansky, 1937). Where RIBs are strong, they may play an important role in species divergence, the maintenance of boundaries among biological species, and the coexistence of closely related species (Coyne & Orr, 2004; Valladares et al., 2015; Weber & Strauss, 2016). Reproductive barriers are typically classified according to when they occur (Baack et al., 2015; Coyne & Orr, 2004). In plants, in addition to zygote formation, pollination is also used as a classification criterion (Baack et al., 2015). Pre‐pollination, pre‐zygotic barriers preclude interspecific pollen transfer through geographical isolation, phenological mismatch, or pollinator differentiation (Baack et al., 2015; Widmer et al., 2009). Some post‐pollination barriers, such as pollen–pistil or pollen tube‐ovule incompatibilities, occur before zygote formation while others, such as the selective abortion of ovules or the unviability of seeds fertilized with heterospecific pollen, are post‐zygotic (Baack et al., 2015; Orr & Turelli, 2001).

Comprehensive reviews, including one narrative (Baack et al., 2015) and two quantitative reviews (Christie et al., 2022; Lowry et al., 2008), of case studies addressing reproductive isolation between pairs of plant species have found that often (i) multiple pre‐ and post‐zygotic/pollination RIBs contribute to the total reproductive isolation between the species, and these are usually redundant, (ii) pre‐ and post‐zygotic RIBs are both asymmetric (i.e., the strength of a RIB is contingent on the species acting as the ovule parent), and (iii) pre‐zygotic barriers are stronger than post‐zygotic barriers (Baack et al., 2015; Christie et al., 2022; Lowry et al., 2008).

The strength of RIBs and their contribution to reproductive isolation have been studied using standard metrics in only a limited number of species pairs (i.e., 89 species pairs were included in the latest review: Christie et al., 2022) relative to the high diversity of seed plants (Baack et al., 2015; Christie et al., 2022). Perhaps of greater concern is the overrepresentation of temperate perennial herbs (mainly belonging to the genera *Iris*, *Helianthus*, *Mimulus*, *Ophrys*, *Pedicularis*, and *Senecio*) in current literature on the topic (Baack et al., 2015; Christie et al., 2022), while other taxa, such as the tropical woody perennials (i.e., shrubs and trees), are represented to a far lesser extent (7% of species pairs included in the review of Christie et al., 2022). This uneven sampling effort in terms of life form and geographic distribution precludes any generalization about reproductive isolation beyond the temperate herbaceous perennials (Baack et al., 2015; Christie et al., 2022).

To our knowledge, the reproductive strength and the relative contribution of pre‐ and post‐zygotic barriers have been assessed with standard metrics in only five pairs of tropical woody perennial species: *Salvia elegans* vs. *S. fulgens* (Cuevas et al., 2018), *Cyrtandra kauaensis* vs. *Cyrtandra longifolia*, *Cyrtandra paludosa* vs. *Cyrtandra platyphylla* (Johnson et al., 2015), *Quercus mongolica* vs. *Q. liaotungensis* (Liao et al., 2019), and *Rhododendron cyanocarpum* vs. *R. delavayi* (Ma et al., 2016). Although the majority (60%) of woody perennial species studied to date have shown the opposite pattern (i.e., post‐zygotic are stronger than pre‐zygotic RIBs) to that depicted in general reviews on the topic (Christie et al., 2022; Lowry et al., 2008), the number of studies available is insufficient to allow us to conclude that woody perennials follow a different pattern than perennial herbs. Although some annuals have a greater frequency of chromosomal rearrangements and a faster rate of molecular evolution than perennials, which may partially account for the observed differences in the evolution of reproductive isolation in some groups of plants (Baack et al., 2015; Gaut et al., 2011), it is probably too early to suggest an underlying mechanism since the very limited availability of data restricts our ability to generalize (Baack et al., 2015). There is thus an urgent need to redirect our efforts toward documenting reproductive isolation in plant species with poorly explored life history traits and distribution, such as those of the tropical woody perennials (Baack et al., 2015; Christie et al., 2022). Although challenging (given their large size and long lifespan), the study of woody perennials holds great promise for improving our understanding of reproductive isolation in plants (Baack et al., 2015; Christie et al., 2022; Stankowski & Ravinet, 2021).

In this sense, an interesting group of plants for the study of RIBs is the Neotropical genus *Cnidoscolus* (Euphorbiaceae; Maya‐Lastra & Steinmann, 2019). *Cnidoscolus* comprises ca. 99 woody species, some of which occur sympatrically across the distribution range of the genus (Maya‐Lastra & Steinmann, 2018). A particularly interesting case for study is presented by *C. aconitifolius* and *C. souzae*, two closely related species that may represent the youngest speciation events in the genus (Maya‐Lastra & Steinmann, 2019; Maya‐Lastra, Unpublished Research). This is important because, despite the theoretical work conducted on the topic (e.g., Seehausen et al., 2014) and correlative evidence (i.e., genetic distance vs. reproductive isolation; e.g., Christie & Strauss, 2018); apart from the case of some polyploids, there is little empirical work in which RIBs appear at the earliest stages of divergence and that addresses how additional RIBs accumulate (Baack et al., 2015; Liao et al., 2019; Widmer et al., 2009). Besides being an early diverging pair of species, *C. aconitifolius* and *C. souzae* are not polyploids (2n = 36; this study). These plant species therefore offer an excellent opportunity to determine which RIBs are developed during the early stages of species divergence when polyploidy is not involved.

**FIGURE 1:**
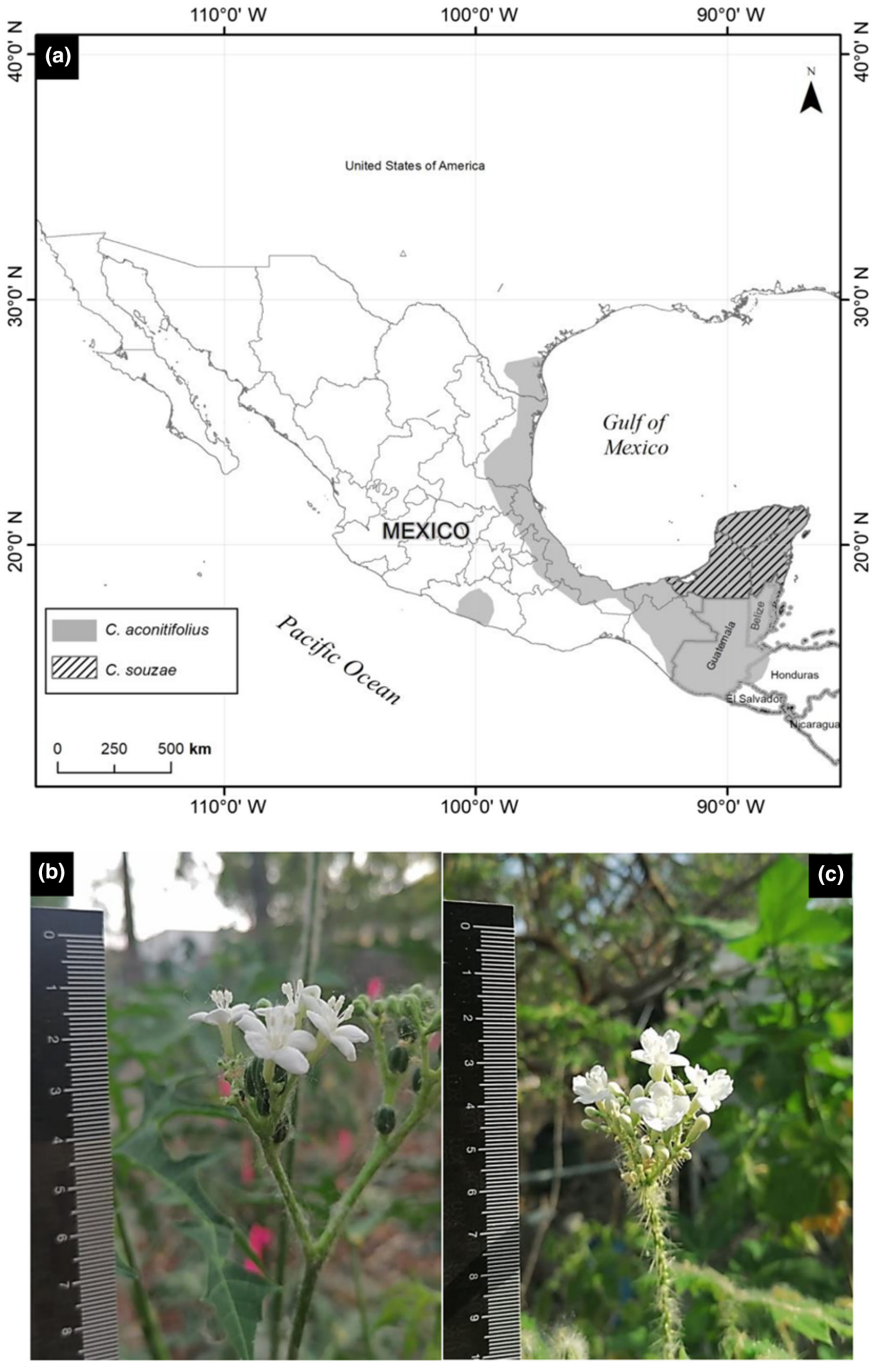
Native distribution range of *Cnidoscolus aconitifolius* and *C. souzae* (a). Photographs of inflorescences with open male flowers of *Cnidoscolus aconitifolius* (b) and *C. souzae* (c). The black rule in B&C shows the length in centimeters.

*Cnidoscolus aconitifolius* and *C. souzae* occur sympatrically on the Yucatan Peninsula, Mexico (Maya‐Lastra & Steinmann, 2019). *Cnidoscolus souzae* is a narrow endemic species, and its entire coarse‐grained distribution range is fully embedded within the wider distribution range of *C. aconitifolius* (Arceo‐Gómez et al., 2016; Ross‐Ibarra & Molina‐Cruz, 2002; Figure 1a). Although allopatric populations of *C. aconitifolius* exist, we experimentally assessed reproductive isolation in sympatry only given that, under this condition, the actual RIBs are far more detectable and play a more important role in preventing gene flow (Coyne & Orr, 2004). A comparison of two independent studies with *C. aconitifolius* (Munguía‐Rosas & Jácome‐Flores, 2020) and *C. souzae* (Arceo‐Gómez et al., 2016) revealed that the flower morphology of these two species is very similar. The flowering phenologies overlap to some extent, and they share their main flower visitors (bees and butterflies). Therefore, contrary to the general trend depicted by the reviews about RIBs in plants (Christie et al., 2022; Lowry et al., 2008), we expected that pre‐zygotic, pre‐pollination barriers between the study species would be weaker than post‐pollination, post‐zygotic RIBs. The specific goals of this research were (i) to assess the total degree of reproductive isolation between *C. aconitifolius* and *C. souzae* in sympatry, (ii) to identify the reproductive isolation barriers and assess their strength and contribution to reproductive isolation, and finally, (iii) to evaluate the level of asymmetry of the RIBs. Asymmetry in RIBs is relevant since it can provide insights into the direction of gene flow and hybrid formation (Keller et al., 2021) in the study species.

## 2 MATERIALS AND METHODS

### 2.1 Study system

*Cnidoscolus aconitifolius* and *C. souzae* are closely related plant species with an estimated mean divergence time of only 0.82 Mya (95% HDP: 2.4 Mya—present, based on the chloroplast intergenic spacer trnL‐trnF; Maya‐Lastra & Steinmann, 2019; Maya‐Lastra, unpublished research). Both species are Neotropical spiny shrubs of up to 5 m in height (Maya‐Lastra & Steinmann, 2018). *Cnidoscolus aconitifolius* inhabits mainly arid and semiarid areas from the south of Texas, through the Gulf of Mexico to Central America (Ross‐Ibarra & Molina‐Cruz, 2002), while *C. souzae* is endemic to the tropical dry forests of the Yucatan Peninsula (Arceo‐Gómez et al., 2016; Kolterman et al., 1984; Figure 1a). Since the coarse‐grained spatial distribution of *C. souzae* completely overlaps that of *C. aconitifolius*, the strength of the geographical barrier is RI_geographic isolation_ = 0 for this species, while for *C. aconitifolius*, RI_geographic isolation_ = 0.72 (calculated using the equation RI_4C_ of Sobel & Chen, 2014. See Section 2.7 below). While plants of the study species are mostly found in mixed populations (with just a few meters in distance among individuals) within the range of co‐occurrence, we have also found some areas where plants of different species present much greater spatial separation (<2 km; Munguía‐Rosas, unpublished research). However, this distance is also within the foraging range of some pollinators (e.g., Beekman & Ratnieks, 2001). Nevertheless, since we used an experimental approach and focused exclusively on reproductive isolation in sympatry, the RIB due to geographic isolation was irrelevant to this study.

Both study species are monoecious and self‐compatible (Arceo‐Gómez et al., 2016; Parra‐Tabla & Herrera, 2010). The male flowers have 10 anthers (five with long and another five with short filaments), while the female flowers have three ovules. The flowers of both species are white and actinomorphic (Figure 1b,c). Although the corollas of the female *C. souzae* flowers are slightly wider (14.44 ± 0.31 mm; hereafter mean values ± 1 SE; ANOVA: *F*_1,63_ = 6.37, *p* < .01, *n* = 65 flowers) and longer (4.95 ± 0.12 mm; ANOVA: *F*_1,63_ = 7.63, *p* < .02, *n* = 65 flowers) than those of *C. aconitifolius* (corolla width = 13.27 ± 0.29 mm, length = 4.53 ± 0.11, *n* = 65 flowers), style length is almost identical in both species (*C. aconitifolius* = 3.42 ± 0.13 mm, *C. souzae* = 3.44 ± 0.12 mm; ANOVA: *F*_1,63_ = 0.02, *p* = .89, *n* = 65 flowers), and the two species are not differentiated in the multivariate morphological space in terms of these variables (Appendix 1a). Male *C. souzae* flowers are also longer (11.63 ± 0.44 cm) than those of *C. aconitifolius* (8.41 ± 0.22; ANOVA: *F*_1,42_ = 48.51, *p* < .01, *n* = 46 flowers), but they do not differ significantly in terms of corolla width (*C. aconitifolius* = 13.41 ± 0.19 mm, *C. souzae* = 13.82 ± 0.35 mm; ANOVA: *F*_1,42_ = 1.25, *p* = .26, *n* = 46 flowers; Appendix 1b). The flowers of both plant species are short‐lived (<1 day) and thus are only accessible to diurnal pollinators; that is, the flowers are abscised before sunset. The flowers of these two species are mainly visited by several species of generalist bees and butterflies (Arceo‐Gómez et al., 2016; Munguía‐Rosas & Jácome‐Flores, 2020). Prior to this study, no information was available regarding hybridization between *C. aconitifolius* and *C. souzae*; however, a few intermediate phenotypes (i.e., plants with spines characteristic of *C. souzae* but petiolar glands typical of *C. aconitifolius*. See Appendix 2) have been found across the Yucatan Peninsula.

### 2.2 Plant material and experimental design

During the year 2018, we collected 100 stem cuttings (40–60 cm in length) from secondary branches of 100 apparently healthy reproductive plants of *C. aconitifolius* and *C. souzae* (*n* = 200 cuttings and plants) in three different populations located in central Yucatan, Mexico: Hunucma (20°59′09.43″ N, 89°49′19.63″ W), Uman (20°53′33.70″ N, 89°45′49.51″ W), and Sierra Papacal (21°07′50.72″ N, 89°46′38.96″ W). The latter two localities belong to the municipality of Merida, while the first belongs to the municipality of Hunucma, adjacent to Merida (ca. 12 km in distance). The vegetation in all three populations was moderately disturbed tropical dry forest. Although plant population size was not measured, it appears to be similar among the sampled locations. Plant density was apparently high (dozens of reproductive adults per hectare in both species) and similar among sites. The minimum distance between donor plants was 10 m. Both species were unambiguously identified in the field based on the morphology of their spines (thin, whitish, and denser in *C. aconitifolius* vs. larger, yellowish, and less dense in *C. souzae*), as well as the petiolar gland (fleshy in *C. aconitifolius* vs. stipitate in *C. souzae*; Appendix 2). Intermediate phenotypes and domesticated forms of *C. aconitifolius* were avoided. The latter was easily identified due to its domestication syndrome (fewer spines, more and bigger leaves, and more succulent stems) and because domesticated plants do not occur in natural vegetation (i.e., domesticated plants rarely set fruit and are thus clonally propagated by humans. Munguía‐Rosas et al., 2019; Munguía‐Rosas & Jácome‐Flores, 2020). Two representative specimens of the collected plant material were deposited at the Herbarium Alfredo Barrera Marín of the University of Yucatan, Campus for Biological and Health Sciences, located in the City of Merida, Yucatan, Mexico (*C. aconitifolius*: UADY‐23474, *C. souzae*: UADY‐23688).

The stem cuttings were planted in 24 L pots filled with a mix of 70% gravel and 30% local soil and kept in a common garden located in the northern region of the municipality of Merida (21°01′18.33″ N; 89°37′33.7″ W) under full sun exposure with watering as required for the first 5 months to reduce mortality. After 1 year, 38 *C*. *souzae* and 43 *C. aconitifolius* plants had rooted and survived. These plants presented high heterogeneity in size and we therefore selected a total of 60 plants (30 per species) with the greatest similarity in terms of height (1–1.5 m) and number of branches (2–4 secondary branches). The selected plants from different species were intercalated in a common garden, with an approximate distance between plants of 1.5 m. The common garden was located within a natural area of co‐occurrence surrounded by vegetation similar to that of the populations of origin. The identity of the pollinators observed in this garden during a previous study (Munguía‐Rosas & Jácome‐Flores, 2020) was similar to that in nearby (ca. 16–35 km in distance) natural populations of *C. aconitifolius* (Parra‐Tabla et al., 2004) and *C. souzae* (Arceo‐Gómez et al., 2016). Since the experimental plants of the two study species shared the same population size and density at the beginning of the experiment, these conditions allowed us to assess RIBs in sympatry while controlling for extrinsic (e.g., above‐ and belowground environment) and intrinsic (e.g., size and age) factors that could act to increase undesired within‐species variation (i.e., error).

### 2.3 Flowering phenology, floral display, and pollinators

Every week, for a period of 1 year (March 2019 to March 2020), we counted the number of inflorescences and open flowers (male and female) in a single randomly chosen inflorescence in each of the 60 plants in the common garden. Furthermore, some buds of the male flowers of the two species were bagged with mosquito netting and, once open, were harvested to quantify the pollen grains of all of the anthers under a light microscope (Leica Microsystems EZ4 HD). In total, 31 and 33 flowers (of 10 and 6 plants) of *C. aconitifolius* and *C. souzae*, respectively, were collected for this pollen count. Pollen production was assessed since it is recognized as an important reward for pollinators in *Cnidoscolus* (Munguía‐Rosas & Jácome‐Flores, 2020). To estimate male and female flower production at the plant level, we multiplied the number of flowers of each sex by the number of inflorescences (Munguía‐Rosas & Jácome‐Flores, 2020). Since male and female flowers live for only 1 day but our sampling was conducted weekly, we also multiplied the estimated number of flowers by seven (Munguía‐Rosas & Jácome‐Flores, 2020). To express pollen production at the plant level, the mean number of pollen grains produced per flower was multiplied by the estimated male flower production for each plant described above. To estimate flowering duration, we counted the number of weeks that each plant was observed presenting open flowers. To obtain the number of days in flower, this value was multiplied by seven. The number of male and female flowers, pollen production, and flowering duration at the plant level (response variables) were compared between plant species (a categorical predictor with two levels: *C. aconitifolius* vs. *C. souzae*) using generalized linear models (GLM; three models in total, one per response variable). Since the response variable was a count in all cases and there was some overdispersion of data, a quasi‐Poisson error distribution was used. As a consequence of the death of some plants during the study, the degrees of freedom were variable.

When the two study species co‐flowered, we observed flower visitors for 20 min in 3–9 flowers per focal plant (mean = 3.12 ± 0.21 flowers per plant). Pollinator observations were conducted on clear days, between 08:00 and 10:00 h in a paired design: flowers of two plants, one of each species (*C. aconitifolius* and *C. souzae*), were observed on the same day. A different pair of plants was observed on each different day and the order of observation of plant species was alternated daily. For standardization, only male flowers were observed in both species. In total, 53 and 72 flowers (15 and 9 plants) of *C. aconitifolius* and *C. souzae* were observed, respectively. The number of effective visitors (those that made physical contact with reproductive organs) was recorded and these were identified to the lowest possible taxonomic level. Pollinator species richness and visiting rate (visits·flower·h^−1^) were calculated on a per‐plant basis (response variables) and compared between species (predictor) using two generalized linear mixed effects models (GLMM). Since the pollinator species richness values are counts and the visiting rate was approximately normally distributed, Poisson and Gaussian error distribution were used, respectively. In both models, an identifier for each plant was included as a random factor.

### 2.4 Pollen–pistil interactions

We conducted reciprocal hand‐pollinations during the spring and summer of 2021; that is, we used conspecific and heterospecific pollen (hereafter, pollen source) to hand‐pollinate female flowers of *C. aconitifolius* and *C. souzae* (hereafter, mother plants) to obtain all of the four possible treatment combinations. We hand‐pollinated virgin female flowers (i.e., those that had been bagged at the bud stage) from 08:00 to 10:00 h with either heterospecific or conspecific pollen. For standardization, pollen from two different plants was used, while pollen from the same plants (i.e., self‐pollination) was avoided. No selection criterion was used for the pollen donors since very few plants (typically 2–6) simultaneously had male flowers with enough pollen available for hand‐pollination. Pollen was gently placed on the stigma until saturation, and the pollinated flower was then labeled and re‐bagged. The flowers were harvested 28–30 h after hand‐pollination, fixed in formalin–acetic acid–alcohol solution, and taken to the laboratory where the pollen grains on the stigma and pollen tubes at the style base were quantified. For this, we dissected the flowers to obtain the styles, which were then softened in 1 N KOH at 65°C for 20 min, rinsed with distilled water, and stained in decolorized aniline blue at 65°C for 20 min (Kearns & Inouye, 1993). The number of pollen tubes was then quantified under a Nikon ECLIPSE E200 fluorescence microscope. In total, 104 flowers were pollinated and harvested: 30 *C. aconitifolius* (17 with conspecific and with 13 heterospecific pollen, *n* = 14 plants) and 74 *C. souzae* (conspecific = 32, heterospecific = 42, *n* = 16 plants). These differences in sample sizes were due to interspecies differences in the availability of flowers during the experiment. The effects of pollen source (conspecific vs. heterospecific), mother plant species (*C. aconitifolius* vs. *C. souzae*), and their interaction, on the proportion of pollen grains that developed pollen tubes and reached the base of the style (response variable), were assessed using a GLM with a binomial error distribution, which is suitable for proportion data.

### 2.5 Fruit set and seed germination

Using the procedure described in the subsection *pollen–pistil interactions*, we hand‐pollinated another 238 flowers: 162 *C. aconitifolius* (conspecific = 73, heterospecific = 89) and 76 *C. souzae* (conspecific = 33, heterospecific = 43). These flowers were revised weekly, and we recorded whether the flowers had aborted or set fruit. Ripened fruits from all treatments were counted and collected, and all the seeds of these fruits were extracted from the capsules using forceps and stored in paper bags until a subsequent assessment of their germination rates. All of the seeds extracted from the fruit were apparently viable (filled and similar in size), with the sole difference that some of the seeds sired from heterospecific pollen were slightly paler.

In September 2021, the stored seeds described above were mechanically scarified (i.e., the elaiosomes were removed and the testa scraped with sandpaper; see Munguía‐Rosas & Álvarez‐Espino, 2022 for details) and disinfected with 0.5% NaOCL for 10 min. The seeds were then placed in Petri dishes with filter paper, watered to field capacity with distilled water, and the dishes sealed with Parafilm. The dishes were left for 10 days at room temperature (28–30°C at night and 30–34°C during the day) with a natural photoperiod (approximately 14 h light and 10 h dark). Seed germination (radicle emergence) was recorded daily until the end of the experiment. In total, 245 seeds were sown: 169 *C. aconitifolius* (conspecific = 129, heterospecific = 40) and 76 *C. souzae* (conspecific = 76, heterospecific = 0). No seeds from heterospecific hand‐pollinations with *C. souzae* as a mother plant were included since the fruit set for this treatment was null.

The effects of pollen source (conspecific vs. heterospecific), mother plant species (*C. aconitifolius* vs. *C. souzae*), and their interaction, on (i) the proportion of flowers that set fruit (response) and (ii) the proportion of germinated seeds (response), were assessed using GLMMs with binomial error distribution.

### 2.6 Chromosome number

To assess chromosome duplication in the hybrids relative to the parent species, we transplanted the seedlings (obtained from treatments described in the subsection *Fruit set and seed germination* above) to 4 L pots and placed them in a plant nursery under complete sun exposure, with watering as required. Seedling mortality was very high and only 14 plants survived 2 weeks after transplantation: six plants of *C. aconitifolius*, four of *C. souzae*, and four hybrids of *C. aconitifolius* × *C. souzae*. Four plants of the parent species, as well as the hybrids, were used to assess the karyotype and chromosomal number from somatic cells at metaphase. These cells were taken from the root tips using the procedure outlined by Rodríguez‐Domínguez et al. (2017). Chromosome photographs were taken with a phase‐contrast microscope (Leica DMRA2, Leica Microsystems) equipped with an Evolution QEI camera (Media‐Cybernetics).

### 2.7 Reproductive isolation metrics

We estimated the strength of the reproductive isolation barriers using the reproductive isolation index (RI), as outlined by Sobel and Chen (2014), for five RIBs in total: three pre‐zygotic barriers, two of which were also pre‐pollination barriers (phenological mismatch and pollinator differentiation) while pollen–pistil incompatibility was a post‐pollination barrier. The remaining two RIBs, fruit set failure and seed unviability, were post‐pollination, post‐zygotic barriers. The RIs for each RIB and mother plant species (*C. aconitifolius* and *C. souzae*) were calculated. RI ranges from −1 to 1, with −1 representing completely disassortative mating, 0 representing random mating, and 1 representing complete reproductive isolation.

Given that phenological and pollinator differentiation affects co‐occurrence, the reproductive isolation for these barriers was calculated with the equation RI_4c_ of Sobel and Chen (2014): RI = 1−$\left(\frac{S}{S+U}\right)$, where S refers to the extent to which study species co‐flowered or the number of shared pollinators, and U refers to the extent to which flowering did not overlap between mother plant species or the number of unshared pollinators. For the remaining barriers, we used the equation RI_4A_ of the same authors: RI = 1–2 × $\left(\frac{H}{H+C}\right),$ where H and C refer to heterospecific and conspecific mating/fitness, respectively. More specifically, for the reproductive barrier due to pollen–pistil incompatibility, H = the proportion of pollen tubes that reach the base of the style after heterospecific hand‐pollinations, and C = the proportion of pollen tubes that reach the base of the style after conspecific hand‐pollinations. For the reproductive barrier due to fruit set failure, H = fruit set from heterospecific hand‐pollinations and C = fruit set from conspecific hand‐pollinations. For the barrier due to seed unviability, H = germination of the seeds sired from heterospecific hand‐pollinations and C = germination of the seeds sired from conspecific hand‐pollinations. For all the RIBs, the expectation of heterospecific pollen transfer/mating under the null model was 0.5 since both species had the same density (See online appendix D in Sobel & Chen, 2014).

To combine individual reproductive barriers and obtain the value of total reproductive isolation (TRI), we used the equation RI_4E_ of Sobel and Chen (2014):

$$\mathrm{TRI}=1-2\times \left(\frac{S_{\text{total}}\times p\left(H|S\right)+U_{\text{total}}\times \left(H|U\right)}{S_{\text{total}}\times p\left(H|S\right)+U_{\text{total}}\times p\left(U|H\right)+S_{\text{total}}\times p\left(C|S\right)+U_{\text{total}}\times p\left(C|U\right)}\right)$$

where *S*_total_ is the product of overlapping flowering and shared pollinators and *U*_total_ is 1−*S*_total_. *U*, *S*, *H*, and *C* are as described above, and *p* indicates conditional probabilities.

We also calculated the absolute contribution (AC_*i*_) of each reproductive barrier (RI_*i*_) as: ${\mathrm{AC}}_{i}={\mathrm{RI}}_{\left[1,i\right]}-{\mathrm{RI}}_{\left[1,i-1\right]},$ where RI_[1,*i*]_ is equivalent to TRI, including all barriers from the first (1) through to the focal barrier (*i*), while RI_[1,*i*−1]_ is the same calculation as for RI_[1,*i*]_ but omits the focal (*i*) barrier.

The asymmetry of each barrier was calculated as the absolute value of the difference of a given barrier between mother plants (Christie et al., 2022; Lowry et al., 2008). Following Scopece et al. (2013), we considered asymmetries greater than 0.25 as significant.

All analyses were performed in R 4.0.3 (R Core Team, 2020), except for the reproductive isolation metrics, which were calculated using the MS Excel template available as online supplementary material in Sobel and Chen (2014). All raw data are available as Appendix S1.

## 3 RESULTS

### 3.1 Flowering phenology, floral display, and flower visitors

At the population level, *Cnidoscolus souzae* flowered throughout the year, while *C. aconitifolius* only flowered from April to September. At the plant level, *C. souzae* on average produced 6.7 times more male flowers, 6.23 times more female flowers, and flowered for 4.69 times longer than *C. aconitifolius* (Table 1). However, the opposite was observed for pollen production; that is, a flower of *C. aconitifolius* produced 1.19 times more pollen on average than *C. souzae* (Table 1).

**TABLE 1:** Production of male and female flowers, flowering duration per plant during a reproductive season in *Cnidoscolus aconitifolius* and *C. souzae* in sympatry.

Variable	*C. aconitifolius*	*C. souzae*	Statistics
Male flowers (Count)	16.94 ± 4.14	114.00 ± 21.38	$\chi_{1}^{2}$ = 1577**
Pollen grains per flower (Count)	296.77 ± 15.30	248.97 ± 14.86	$\chi_{1}^{2}$ = 7.53**
Female flowers (Count)	5.56 ± 1.96	34.65 ± 4.50	$\chi_{1}^{2}$ = 459**
Flowering duration (Days)	19.83 ± 1.72	93.13 ± 10.14	$\chi_{1}^{2}$ = 1014**
Pollinator richness (Species No.)	2.27 ± 0.44	0.96 ± 0.19	$\chi_{1}^{2}$ = 10.38**
Pollinator visiting rate (Visits·flower·h^−1^)	6.60 ± 1.61	1.57 ± 0.32	$\chi_{1}^{2}$ = 10.38**

*Note*: The mean number of pollen grains produced by a single flower is also included. The statistics refer to comparisons between the species.

** *p* < .01.

Thirteen different insect species visited the two mother plant species: five bee species, five butterfly species, one species of ant, one dipteran species, and a species of beetle. Ten insect species visited the flowers of *C. aconitifolius* and eight species visited those of *C. souzae* (Table 2). The two plant species shared five pollinator species, while five and three insect species exclusively visited the flowers of *C. aconitifolius* and *C. souzae*, respectively (Table 2). At the plant level, the flowers of *C. aconitifolius* received significantly more diverse visitors (2.27 ± 0.44 pollinator species), and at a faster rate (6.60 ± 1.61 visits·flower·h^−1^), than those of *C. souzae* (0.96 ± 0.19 pollinator species; 1.57 ± 0.32 visits·flower·h^−1^; Table 1).

**TABLE 2:** Floral visitors of *Cnidoscolus aconitifolius* and *C. souzae* in sympatry.

Floral visitor			Plant species	
Order	Family	Species	*C. aconitifolius*	*C. souzae*
Coleoptera		Coleoptera sp1		+
Diptera	Bombyliidae	Bombyliidae sp1	+	
Hymenoptera	Apidae	*Apis mellifera*	+	+
	Formicidae	Camponotus sp1		+
	Halictidae	Agapostemon sp1	+	
		Halictidae sp1	+	+
		Halictidae sp2	+	
		Halictidae sp3	+	
Lepidoptera	Esperiidae	Esperiidae sp1	+	+
		Esperiidae sp2	+	+
		Esperiidae sp3		+
	Lycaenidae	Lycaenidae sp1	+	+
	Nymphalidae	Heliconius sp1	+	
Total		13	10	8

*Note*: A complete list of the recorded visitors is shown in the species column. Species that visited each plant species are indicated with +. Cells in light and dark gray indicate exclusive and shared visitor species, respectively. The order and family of each visitor are also shown.

### 3.2 Pollen–pistil interactions

**FIGURE 2:**
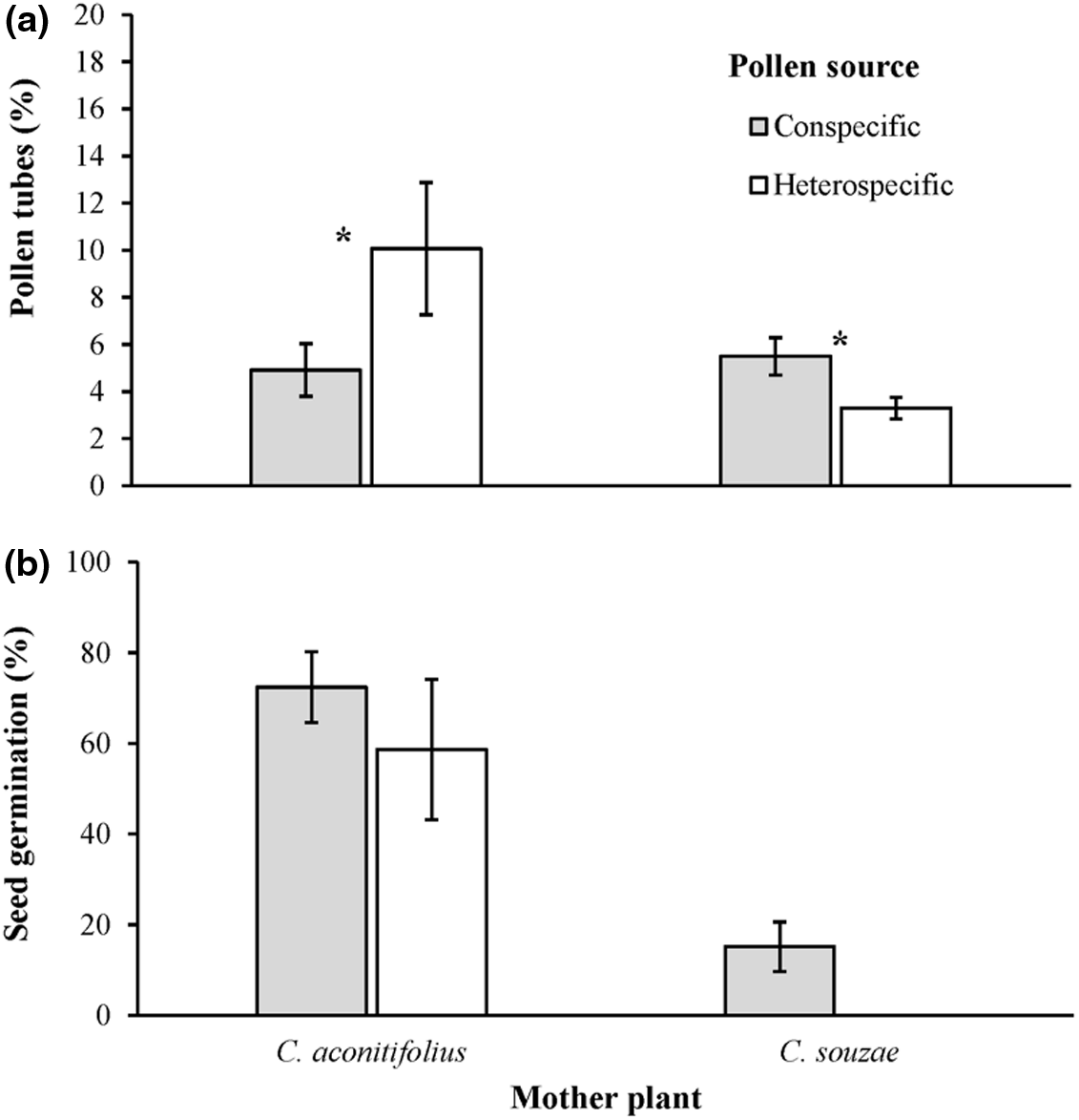
Pollen tubes that reached the base of the style (Pollen tubes; a) and seed germination (b) of *Cnidoscolus aconitifolius* and *C. souzae* pollinated by hand with conspecific and heterospecific pollen. The bars show mean values ± 1 SE. The asterisk indicates statistically significant differences between pollen sources within each species.

The percentage of pollen grains that developed pollen tubes and reached the base of the style was 1.67 times greater in *C. aconitifolius* (7.15 ± 1.43%) than in *C. souzae* (4.26 ± 0.45%; $\chi_{1}^{2}$= 9.52, *p* < .01), as mother plants. This variable was also significantly affected by the pollen source ($\chi_{1}^{2}$ = 30.25, *p* < .01) and the interaction pollen source × mother plant species ($\chi_{1}^{2}$ = 4.15, *p* = .04); that is, the percentage of grains that developed pollen tubes in flowers that were pollinated with conspecific pollen (5.29 ± 0.64) was 1.07 times greater than in those pollinated with heterospecific pollen (4.93 ± 0.84). However, the significant interaction suggested that the effect of pollen source was contingent on the identity of the mother plant; that is, in the pistil of *C. aconitifolius*, a greater percentage of heterospecific pollen (10.07 ± 2.81%) developed pollen tubes and reached the base of the style than was the case for conspecific pollen (4.91 ± 1.12%), while the opposite was observed in *C. souzae* (conspecific: 5.49 ± 0.80%; heterospecific: 3.30 ± 0.46%; Figure 2a).

### 3.3 Fruit set and seed germination

Regardless of the mother plant species, fruit set was 12.59 times greater in flowers that were hand‐pollinated with conspecific pollen (94.71 ± 3.18%) than in those hand‐pollinated with heterospecific pollen (7.52 ± 5.31%; $\chi_{1}^{2}$ = 37.03, *p* < .01). Differences in terms of fruit set between mother plant species (*C. aconitifolius* = 55.71 ± 10.73%, *C. souzae* = 43.14 ± 11.79%; $\chi_{1}^{2}$ = 0.54, *p* = .46), as well as the effect of the interaction pollen source × mother plant species on the same variable ($\chi_{1}^{2}$ = 0.01, *p* = .99), were not statistically significant. Thus, the fruit set of flowers hand‐pollinated with conspecific pollen was very high (*C. aconitifolius* = 97%; *C. souzae* = 88%), but very low (*C. aconitifolius* = 14.28 ± 9.82%) or null (*C. souzae*) in those hand‐pollinated with heterospecific pollen in both mother plant species (Table 3).

**TABLE 3:** Fruit set of hand‐pollinated flowers of *Cnidoscolus aconitifolius* and *C. souzae*, using two different pollen sources: conspecific versus heterospecific pollen.

Mother plant	Pollen source	Fruit set (%)
*C. aconitifolius*	Conspecific	97.14 ± 2.85ª
	Heterospecific	14.28 ± 9.81^b^
*C. souzae*	Conspecific	88.88 ± 8.24
	Heterospecific	0

*Note*: Data are mean values ± 1 SE. Different superscript letters indicate statistically significant differences between pollen sources (*p* < .05). The effect of conspecific versus heterospecific pollen sources on fruit set was not statistically tested for *C. souzae* since this was null.

Germination of seeds sired from flowers hand‐pollinated with conspecific pollen was 4.77 times greater in *C. aconitifolius* (72.42 ± 7.84%) than in *C. souzae* (15.16 ± 5.47%), a difference that was statistically significant ($\chi_{1}^{2}$ = 29.13, *p* < .01). Germination of seeds sired from flowers hand‐pollinated with heterospecific pollen (50.63 ± 15.50%) did not differ statistically from that of seeds sired from flowers hand‐pollinated with conspecific pollen ($\chi_{1}^{2}$ = 2.21, *p* = .14) in *C. aconitifolius* (Figure 2b).

### 3.4 Chromosome number

**FIGURE 3:**
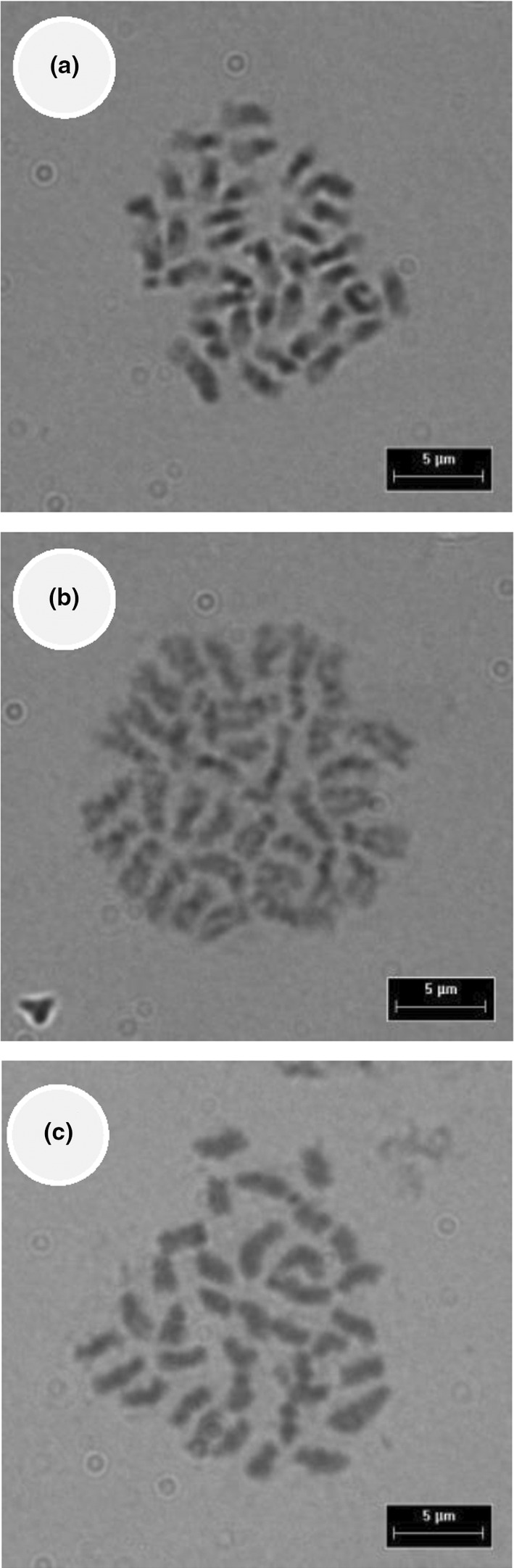
Karyotypes of *Cnidoscolus aconitifolius* (a) *C. souzae* (b) and hybrids *C. aconitifolius* × *C. souzae* (c).

*Cnidoscolus aconitifolius*, *C. souzae*, and the hybrids *C. aconitifolius* × *C. souzae* were all diploid, and the chromosome number was 2n = 36 (Figure 3).

### 3.5 Reproductive isolation metrics

While *Cnidoscolus souzae* reached complete reproductive isolation (TRI = 1), this condition was only partial for *C. aconitifolius* (*TRI* = 0.88; Table 4). Maximum cumulative reproductive isolation was reached earlier in *C. souzae* (reproductive isolation accumulated until the fruit set stage) than in *C. aconitifolius* (reproductive isolation accumulated until the seed germination stage; see cumulative RI in Table 4). The asymmetry in total isolation between *C. aconitifolius* and *C. souzae* was 0.22 (Table 4). For both mother plant species, two post‐pollination, post‐zygotic barriers (fruit failure set and seed unviability) were the strongest isolation barriers (Table 4). However, the strength and asymmetry of seed unviability could not be calculated for *C. souzae* as a mother plant because no seed was sired with heterospecific pollen (Table 4). Interestingly, pollen–pistil incompatibility was negative for *C. aconitifolius*. Asymmetry of RIBs was >0.25 in most of the barriers evaluated in this study: phenological mismatch, pollen–pistil incompatibility, and fruit set failure. Of these barriers, pollen–pistil incompatibility presented the greatest asymmetry (Δ = 0.58; Table 4).

**TABLE 4:** Strength of reproductive barriers, cumulative reproductive isolation, and absolute contribution to reproductive isolation and total reproductive isolation between *Cnidoscolus aconitifolius* and *C. souzae* in sympatry.

Metric	Barrier type	Barrier	Mother plant		Δ
			*C. aconitifolius*	*C. souzae*	
RIB strength	Pre‐pollination Pre‐zygotic	Phenological mismatch	0.00	0.50	0.50
		Pollinator differentiation	0.50	0.38	0.12
	Post‐pollination Pre‐zygotic	Pollen–pistil incompatibility	−0.34	0.24	0.58
	Post‐pollination Post‐zygotic	Fruit set failure	0.74	1.00	0.26
		Seed unviability	0.10		
Cumulative RI	Pre‐pollination Pre‐zygotic	Phenological mismatch	0.00	0.50	
		Pollinator differentiation	0.50	0.69	
	Post‐pollination Pre‐zygotic	Pollen–pistil incompatibility	0.33	0.76	
	Post‐pollination Post‐zygotic	Fruit set failure	0.86	1.00	
		Seed unviability	0.88	1.00	
Total RI			0.88	1.00	0.22
Absolute contribution to RI	Pre‐pollination Pre‐zygotic	Phenological mismatch	0.00	0.50	
		Pollinator differentiation	0.50	0.19	
	Post‐pollination Pre‐zygotic	Pollen–pistil incompatibility	−0.17	0.07	
	Post‐pollination Post‐zygotic	Fruit set failure	0.52	0.23	
		Seed unviability	0.02	0.00	

*Note*: The asymmetries between species for each barrier and metric are also shown (Δ). RIB, Reproductive isolation barrier; RI, Reproductive isolation. The strength of RI and Δ was not calculated for seed unviability in *C. souzae* because no seed was sired from heterospecific hand‐pollinations.

One pre‐pollination, pre‐zygotic RIB, and one post‐pollination, post‐zygotic RIB made the greatest absolute contribution to reproductive isolation in both mother plant species (Table 4). Specifically, pollinator differentiation (AC = 0.50) and fruit set failure (AC = 0.52) in *C. aconitifolius*, and phenological mismatch (AC = 0.50) and fruit set failure (AC = 0.23) in *C. souzae* made the greatest relative contributions (Table 4). Pollen–pistil incompatibility also presented negative values for *C. aconitifolius* and a low value (0.07) for *C. souzae* in terms of the absolute contribution to reproductive isolation. Phenological mismatch made a null absolute contribution to reproductive isolation (AC = 0) in *C. aconitifolius* (Table 4).

## 4 DISCUSSION

In this study, we have shown that a post‐zygotic reproductive barrier (fruit set failure) was the strongest barrier in two species of tropical shrubs of the genus *Cnidoscolus*. We have also shown that most RIBs assessed in this system were significantly asymmetrical and that the level of asymmetry exhibited in pre‐zygotic RIBs tends to be greater than in post‐zygotic RIBs. While reproductive isolation from *C. aconitifolius* to *C. souzae* was complete, it was only partial (88%) in the opposite direction. Some of these findings are in contrast to the previously suggested pattern that pre‐zygotic RIBs are stronger (Baack et al., 2015; Christie et al., 2022; Lowry et al., 2008) but equally asymmetrical (Christie et al., 2022) in plants.

As stated above, contrary to the previous suggestions that pre‐zygotic reproductive barriers are stronger (Baack et al., 2015; Christie et al., 2022; Lowry et al., 2008), we have shown a post‐zygotic barrier to be the strongest barrier in two closely related *Cnidoscolus* species under sympatry. It is known that heterospecific pollen deposition and heterospecific pollen germination can increase with phylogenetic relatedness, suggesting that the pollen–pistil recognition system may be phylogenetically conserved (Streher et al., 2020). Given that the study species are closely related, this high phylogenetic relatedness may at least partially explain why pollen–pistil incompatibility was the weakest pre‐zygotic barrier. Moreover, similarities between the study species in terms of flower morphology and floral biology (Arceo‐Gómez et al., 2016; Munguía‐Rosas & Jácome‐Flores, 2020) could explain why other pre‐pollination, pre‐zygotic barriers, such as pollinator differentiation (RI: 0.38–0.50), were also relatively weak in this pair of *Cnidoscolus* species. Thus, our findings have added to the known exceptions (e.g., Costa et al., 2007; Jewell et al., 2012; Johnson et al., 2015; Liao et al., 2019) to the previously depicted pattern regarding the greater strength exhibited by pre‐zygotic barriers (Baack et al., 2015; Lowry et al., 2008). Although similar results have been found with most of the tropical woody perennials studied so far (*S. fulgens* (Cuevas et al., 2018), *Cyrtandra kauaiensis*, *Cyrtandra longifolia*, *Cyrtandra platyphylla* (Johnson et al., 2015), *Q. mongolica*, and *Q. liaotungensis* (Liao et al., 2019)), an important proportion (40%) of the studied species exhibited the opposite pattern (*Cyrtandra paludosa* (Johnson et al., 2015), *R. cyanocarpum*, *R. Delavayi* (Ma et al., 2016), and *S. elegans* (Cuevas et al., 2018)). It is therefore crucial to increase the number of studies of woody perennials in the tropics to assess whether or not this group of plants conforms to the pattern described above.

On the contrary, our study concurs with previous studies regarding the relatively high prevalence of asymmetry, particularly in pre‐zygotic barriers (Lowry et al., 2008; but see Christie et al., 2022). The majority of RIBs for which asymmetry was assessed in this study (four of five) presented values greater than 0.25. This is important because the asymmetries of some barriers are indicative of the direction of gene flow and hybridization (Keller et al., 2021; Tiffin et al., 2001). Although slightly lower than 0.25, asymmetry in total reproductive isolation was also important (Δ = 0.22), suggesting that heterospecific pollen transfer and hybridization potential is greater in the direction *C. souzae* to *C. aconitifolius*. The fact that the reproductive isolation of *C. aconitifolius* is incomplete (TRI = 0.88), and that some hybrids with *C. souzae* were obtained (using *C. aconitifolius* as the ovule parent), may represent a reproductive cost for *C. aconitifoliu*s when occurring in sympatry with, and in similar densities to, its congeneric species.

Among pre‐pollination barriers, pollinator differentiation was the strongest (RI = 0.50) and also made the greatest absolute contribution to reproductive isolation (AC = 0.50) among all of the pre‐pollination RIBs in the direction *C. souzae* to *C. aconitifolius*. This is an unexpected result given the high similarity of flower morphology and biology that exists between these two species (Arceo‐Gómez et al., 2016; Parra‐Tabla & Herrera, 2010). *Cnidoscolus aconitifolius* was visited more intensively by a richer assemblage of pollinators, 50% of which were not shared with *C. souzae*. This greater visitation may be due to the higher pollen production, which is an important reward for flower visitors in *C. aconitifolius* (Munguía‐Rosas & Jácome‐Flores, 2020). The differences in the assemblage of pollinators could be due to the larger corollas presented by *C. souzae* that probably acted to exclude short‐tongued pollinators. This is in line with our observation that more bee species (which are short‐tongued insects) visited *C. aconitifolius* than *C. souzae*. The fact that floral tube length has previously been identified as a trait that narrows floral visitor assemblage also supports this notion (e.g., Herrera, 1996; Moré et al., 2007). An important lesson learned from our study is that flower morphology is not a reliable predictor of the strength of pollinator differentiation as a RIB. A wide variety of strategies may prevent heterospecific transfer, even in plant species with similar morphologies (Moreira‐Hernández & Muchala, 2019). One factor that remains to be determined is the quantity of heterospecific pollen that is transferred by the pollinators. Despite our efforts, the identification of pollen from the study species was not feasible since the pollen grains of *C. aconitifolius* and *C. souzae* are nearly identical in size (Diameter ≈ 50 μm), and their exines are morphologically undistinguishable (See Appendix S2). Further research using modern molecular techniques may serve to resolve this issue (Ouyang & Zhang, 2018).

Regarding pollen–pistil incompatibility, this reproductive barrier was negative (RI = −0.34) for *C. aconitifolius* but positive for *C. souzae* (RI = 0.24), meaning that a greater proportion of heterospecific pollen reached the base of the styles of *C. aconitifolius* compared with conspecific pollen. This result (negative RI for pollen–pistil incompatibility) is not uncommon in the literature and is usually attributed to differences in style length or the degree of self‐compatibility (e.g., Costa et al., 2007; Ramírez‐Aguirre et al., 2019; Scopece et al., 2013). However, in our system, style length did not differ significantly (this study) and the study species are self‐compatible (Arceo‐Gómez et al., 2016; Parra‐Tabla et al., 2004; Parra‐Tabla & Herrera, 2010). Another factor must therefore account for the differences in pollen performance in different mother plants in our system. One possibility that merits further exploration is the differential limitation of the growth of tubes of conspecific and heterospecific pollen by the internal tissue of the styles (Broz & Bedinger, 2021).

The percentage of conspecific pollen that germinated and developed pollen tubes in *C. souzae* as the mother plant (ca. 5%) was substantially lower than that previously reported in natural populations for this species (11%–46%; Arceo‐Gómez et al., 2016). However, pollen tubes have been quantified at different levels of the style: While we counted these tubes at the base of the style, the authors of the previous study counted them in the first 0.5 cm of the style (approximately the first half of a squashed style considering stigma lobes) and this may explain the differences. However, we consider that counting pollen tubes at the style base is more appropriate if the interest is in pollen–pistil incompatibility since all the barriers presented in the style have probably acted at this level.

Although abortion of fruit from heterospecific hand‐pollinations was high, some fruits (14%) did ripen and produce viable seeds (germination ≈ 70%) in *C. aconitifolius*. Thus, there is some chance (≈12%) of producing hybrids, but only with *C. aconitifolius* as the ovule parent. When the hybrids are unfit, this can represent a reproductive cost to one or both parent species, which may ultimately lead to the evolution of early‐acting barriers (reinforcing) that reduce gamete discounting via natural selection or local extinction of the most vulnerable parent species (Hopkins, 2013). Since the study species are at the early stage of divergence, perhaps the time elapsed has been insufficient to develop full reproductive isolation in *C. aconitifolius*. Another possibility is that the presence of hybrids has no significant impact on parent species due to their low incidence, as has been observed in other systems (Rieseberg & Carney, 1998). However, an adequate assessment of the hybridization cost for parent species requires exploration of the performance and fertility of hybrids in the field, aspects that could not be addressed in this study due to the low survivorship of both the hybrids and parent plants at the early stages. The fact that hybrids were obtained and that the chromosome number was unaltered during hybridization (2n = 36 for parent species and hybrids) leads us to consider that these hybrids could mate with parent species and produce some seeds, an issue that should be explicitly tested in future research.

In conclusion, reproductive isolation between *C. aconitifolius* and *C. souzae* is complete in the direction *C. aconitifolius* to *C. souzae*, and high but partial in the opposite direction. In both species, pre‐ and post‐pollination/zygotic barriers are involved, with one example of the latter (fruit set failure) being the strongest. Most barriers are asymmetrical, and the level of asymmetry was stronger in the pre‐zygotic barriers. Furthermore, the asymmetry of total RI predicted the direction of heterospecific pollen flow and hybrid formation. Although the study of reproductive barriers in woody perennials is often logistically challenging (owing to their large size and long lifespan), more studies with these plant species are required to determine whether pre‐zygotic barriers are consistently stronger in plants that present this life form.

## AUTHOR CONTRIBUTIONS

**Víctor Parra‐Tabla:** Data curation (lead); methodology (equal); visualization (lead); writing – original draft (equal); writing – review and editing (equal). **Miguel A. Munguía‐Rosas:** Conceptualization (lead); formal analysis (lead); investigation (lead); writing – original draft (lead); writing – review and editing (equal). **José M. Rodríguez‐Domínguez:** Methodology (equal); validation (equal); writing – original draft (supporting); writing – review and editing (equal).

## CONFLICT OF INTEREST STATEMENT

Authors have no competing interests to declare.

## Supporting information


**Appendix S1**.

**Appendix S2**.

## Data Availability

All raw data are available in the Appendix S1.

## APPENDIX 1

### 1.1

Scatter plot of morphological traits of female (a) and male flowers (b) of *C. aconitifolius* and *C. souzae*. In the case of (a), the axes are the two first principal components derived from a PCA with three morphological variables: corolla length and width, and style length.

## APPENDIX 2

### 2.1

Leaf and petiole of *Cnidoscolus aconitifolius* (a), of an intermediate phenotype (presumably of a hybrid plant) between *C. aconitifolius* × *C. souzae* (b), and of *C. souzae* (c). The insert image next to each leaf is a magnified view of the petiolar gland.
