# Supplementary figures and images for: Partial and asymmetrical reproductive isolation between two sympatric tropical shrub species: Cnidoscolus aconitifolius and C. souzae (Euphorbiaceae)
Source: Ecol Evol. 2023 Dec 11;13(12):e10801. doi: 10.1002/ece3.10801 (PMC10714054; doi:10.1002/ece3.10801)

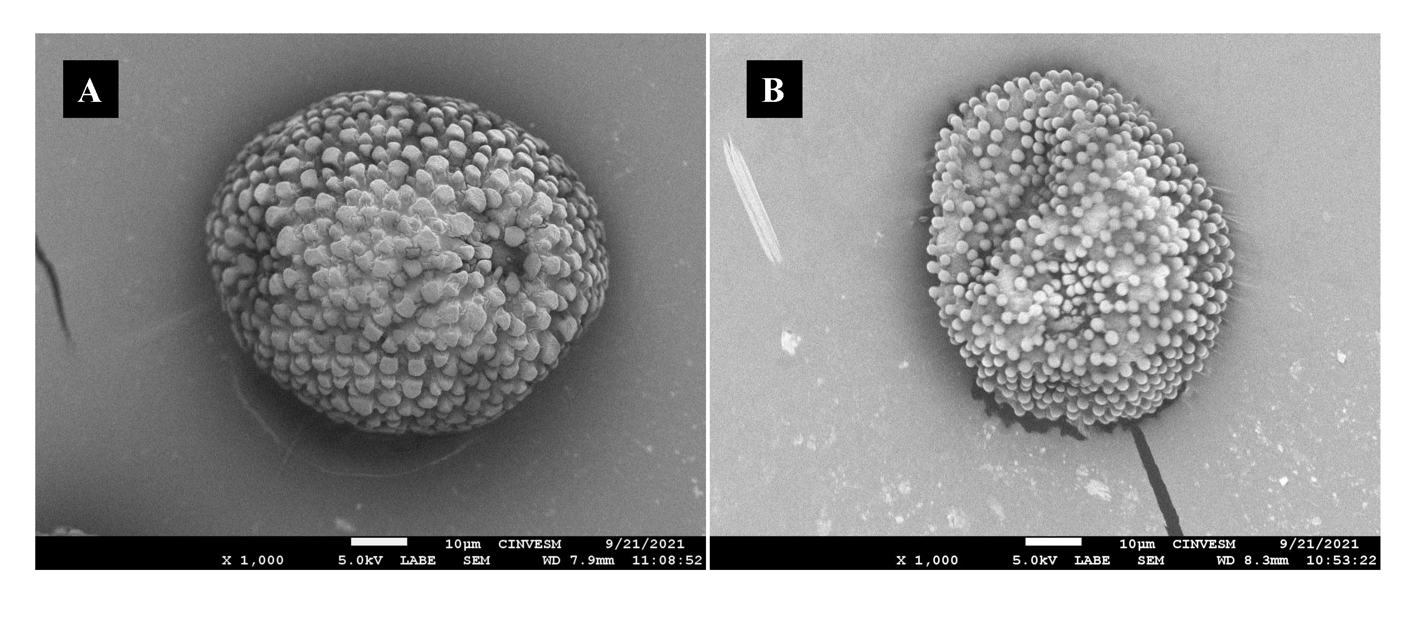

Supplement: Supplementary file 2 — Appendix S2. [file ECE3-13-e10801-s001.tiff]
